# Supplementary material for: Structure of Escherichia coli cytochrome bd-II type oxidase with bound aurachin D
Source: Nat Commun. 2021 Nov 11;12:6498. doi: 10.1038/s41467-021-26835-2 (PMC8585947; doi:10.1038/s41467-021-26835-2)
Supplement: Supplementary file 1 — Supplementary Information [file 41467_2021_26835_MOESM1_ESM.pdf]

Supplementary Information

Structure of *Escherichia coli* cytochrome *bd*-II type oxidase with bound aurachin D

A. Grauel, J. Kägi et al.

3 Supplementary Tables

9 Supplementary Figures

Supplementary Table 1. Cryo-EM parameters and statistics.

Data collection:	
Voltage (kV)	300
Electron exposure (e^-/A^2)	78.9
magnification	75,000
Recording mode	counting
Movie frames	47
Defocus range (µm)	1.4–2.4
Number of collected movies	1836
Map parameters	
Final particles (no.)	125,497
Symmetry imposed	C2
Map resolution (Å)	3.0 Å
FSC threshold	0.143
Map resolution range (Relion local res.) (Å)	2.74-5.7
Map sharpening B factor (Å)	-100
Model refinement	
Initial model used (PDB code)	6RX4
Model composition	
Non-hydrogen atoms	14882
Protein residues	1816
ligands	10
B factors ($Å^2$)	94
Protein	95
ligand	86
Validation	
MolProbity score	1.48
Clashscore	9.07
Poor rotamers (%)	0.26
R.m.s. deviations	
Bond length (Å)	0.008
Bond angles (°)	0.763
Ramachandran plot	
Favored (%)	98.00
Allowed (%)	2.00
Outliers (%)	0.00

Supplementary Table 2. Oligonucleotides used in this work. Homologous sequences are marked bolt. The sequences are given in 5´→3´direction.

pET28b(+) fwd	**GTATGGCTGGACCCGCGTCTGAAAAGTTAA**ATTGGCGAATGGGACGCGCCCTGTAGCG
pET28b(+) rev	GCCAGCGCGATAAATCAATGACATCCCACATGCGCACT**CCTGCTGCCCATGGTATATCTCCTTCTTAAAGTTAAAC**
appC fwd	**GTTTAACTTTAAGAAGGAGATATACCATGGGCAGC**AGGAGTGCGCATGTGGGATGTCATTGATTTATCGCGCTGGC
appX rev	**CGCTACAGGGCGCGTCCCATTCGCCAAT**TTAACTTTTCAGACGCGGGTCCAGCCATAC
appC$_{his}$ fwd	CCGACGCAGCAACAGGGGGGCAGCGGTCATCATCATCATCACCATTAAAGGAGAAAATCATG
appB rev	GCCGACGAACCAAAGTAAATACCACATTGTTTCTGCTCCTTAGTACAACTCGTTTTCGTTACGGCG
cydA G100A fwd	CGCCGCTAGCAATCGAAGCGCTGATGGCCTTCTTCCTCG
cydA G100A rev	CGAGGAAGAAGGCCATCAGCGCTTCGATTGCTAGCGGCG
appC D239N fwd	CTCTGGCGATTATCGGTACCCTGCAACTCGGGAACAGTTCTGCGTATGAAGTCGCGCAAGTACAAC
appC D239N rev	GTTGTACTTGCGCGACTTCATACGCAGAACTGTTCCCGAGTTGCAGGGTACCGATAATCGCCAGAG

Supplementary Table 3. Mediators used in the electrochemical titrations to determine the redox potential of the heme groups.

Compound	E^0, mV (vs SHE)	Solvent	Producer
ferrocenylmethyltrimethylammoniumiodide	806	ethanol	Strem chemicals
1,1’ - ferrocenedicarboxylic acid	635	ethanol	Fluka
potassiumhexacyanoferrate(II) trihydrate	411	water	Riedel-de-Haen
1,1’ - dimethylferrogene	332	ethanol	Aldrich
quinhydrone	269	ethanol	Fluka
tetrachloro — 1,4 -benzoquinone (*p*-Chloranil)	271	acetone	Aldrich
N,N,N’,N’ — tetramethyl-*p*-phenylenediamine dihydrochloride	261	water	Fluka
2,6-dichlorophenolindophenol sodium salt hydrate	208	ethanol	Biochemika
hexaammineruthenium(III)chloride	191	water	Aldrich
anthraquinone-2-sulfonic acid sodium salt	176	water	Aldrich
1,4 naphthoquinone hydrate	136	ethanol	Aldrich
anthraquinone	91	ethanol	Aldrich
5-hydroxy-1,4-naphtoquinone	41	ethanol	Aldrich
duroquinone	1	ethanol	Sigma
menadione	-21	acetone	Sigma
2-hydroxyl-1,4-naphthoquinone	-134	ethanol	Sigma
9,10-antraquinone-2,6-dizulfonic acid disodium salt	-234	ethanol	Sigma
Neutral Red	-316	ethanol	Sigma-Aldrich
methyl viologen dichloride hydrate	-429	water	Aldrich

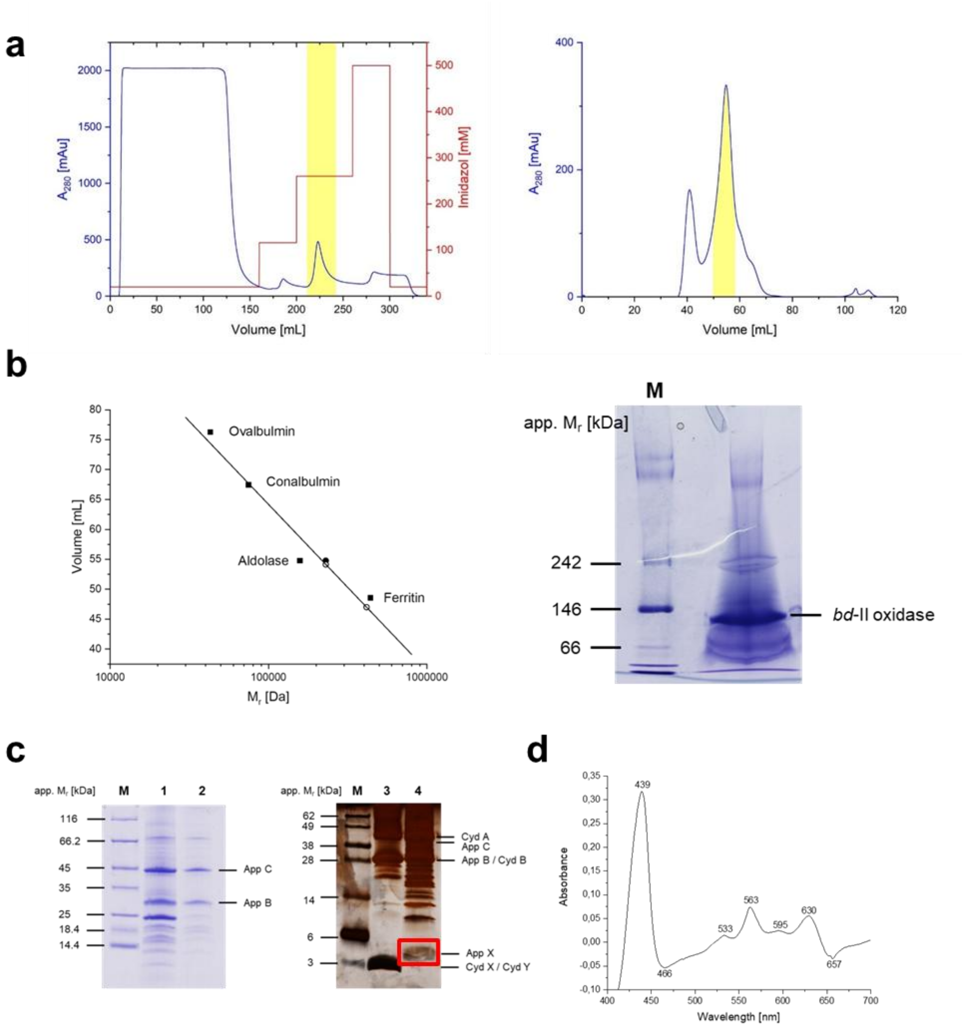

Supplementary Figure 1. Preparation of *bd*-II from *E. coli* strain BL21* Δ*cyoI* pET28b(+) *appC$_h$BX*.

a Elution profiles from HisTrap FF (left) and HiLoad 16/60 Superdex 200 pg (right). The absorbance at 280 nm is shown in blue, the imidazole concentration in red. The pooled fractions are indicated by the yellow area. **b** The preparation of the *bd*-II oxidase is a monomer. Calibration curve of the Superdex 200 column (left) with the elution volumes of the marker proteins (n=1) shown as filled squares. The predicted elution volumes of *bd*-II monomer and dimer are shown as empty circles. The elution volume of the preparation perfectly matches that of the *bd*-II monomer (identical peak position obtained with 2 preparations; filled circle). BN-PAGE of the preparation (right) with marker proteins loaded on lane M. The preparation of *bd*-II oxidase shows an apparent molecular below 146 kDa in accordance with its molecular mass of 105 kDa as derived from the DNA sequence. No distinct band around 210 kDa is detectable. **c** SDS-PAGE of the *bd*-II preparation. Lane 1 shows the protein pattern of the eluate from affinity and lane 2 that of the eluate from size-exclusion chromatography on a 10% gel after Coomassie Blue staining. Lane 3 shows the protein pattern of a preparation of *bd*-I and lane 4 that of the *bd*-II preparation, both after size-exclusion chromatography on silver stained 16% gel. M refers to the pattern of marker proteins. The gels are representatives of 3 different gels. **d** UV-vis (dithionite-reduced minus air-oxidized) difference spectrum of the *bd*-II preparation. The respective peak absorbance wavelengths are indicated.

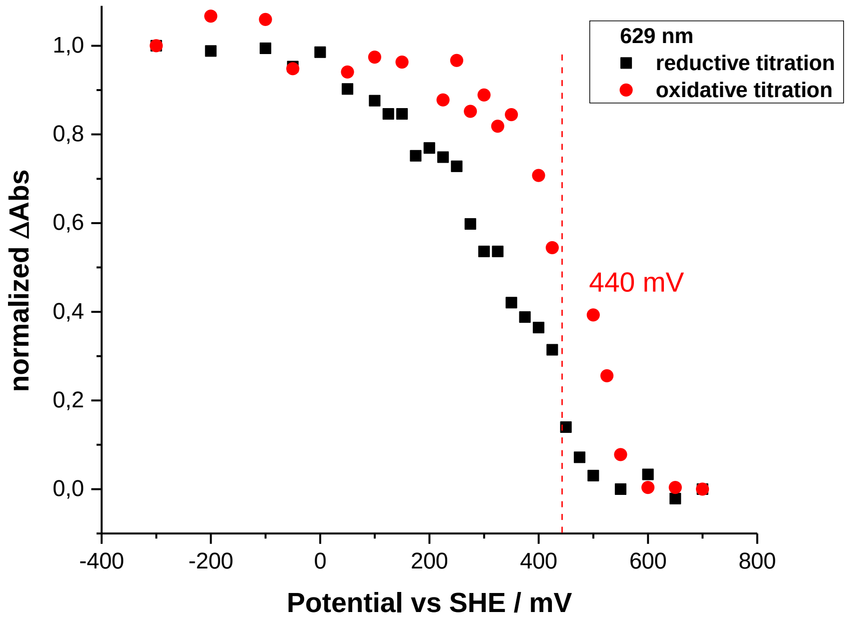

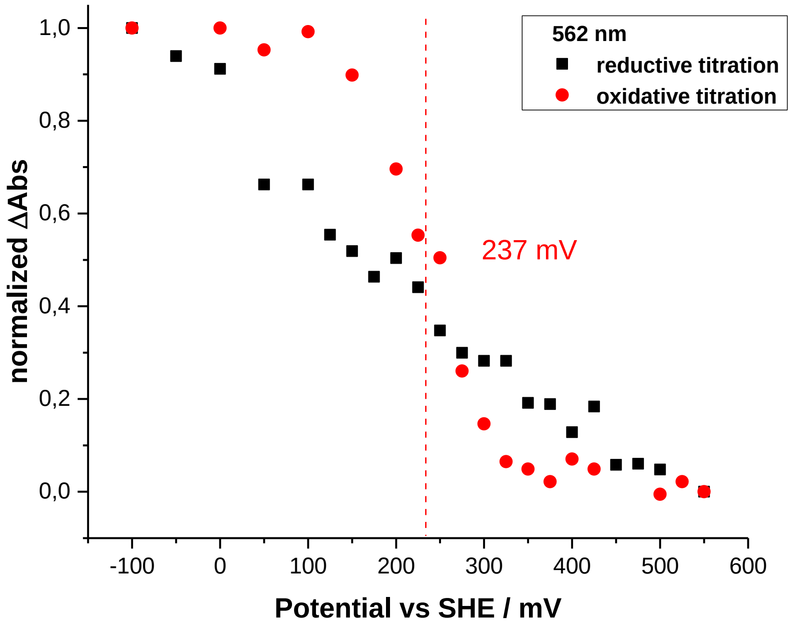

Supplementary Figure 2. Reductive (black) and oxidative (red) electrochemical titration of the absorbance at 629 nm.

The redox potential resulting from reductive and oxidative titration at 629 nm was determined to 440 mV, indicating the lack of processes coupled to the electron transfer reaction. The titration recorded at 562 nm resulted in a redox potential of 237 mV.

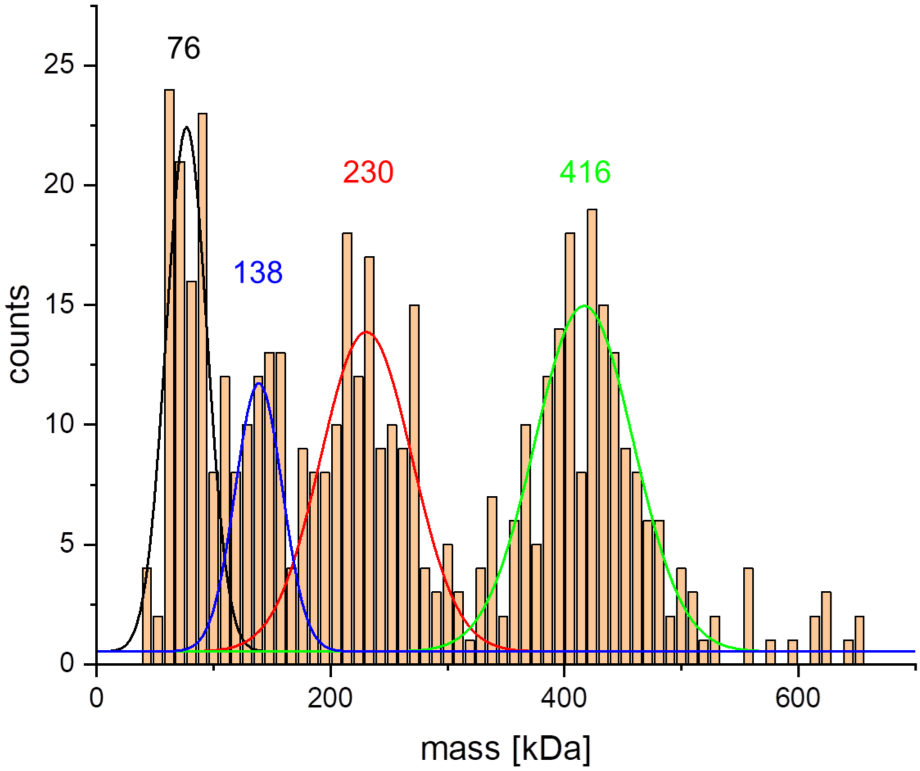

Supplementary Figure 3. Mass distribution for 12 mM *bd*-II as determined by mass photometry[31].

The 416 kDa mass (green curve) was interpreted as *bd*-II dimer and the 230 kDa mass (red) was attributed to the *bd*-II monomer, both with bound detergent. The binding event at 138 kDa (blue) might derive from AppC and/or AppB with bound detergent and that at 76 kDa (black) from the LMNG micelle.

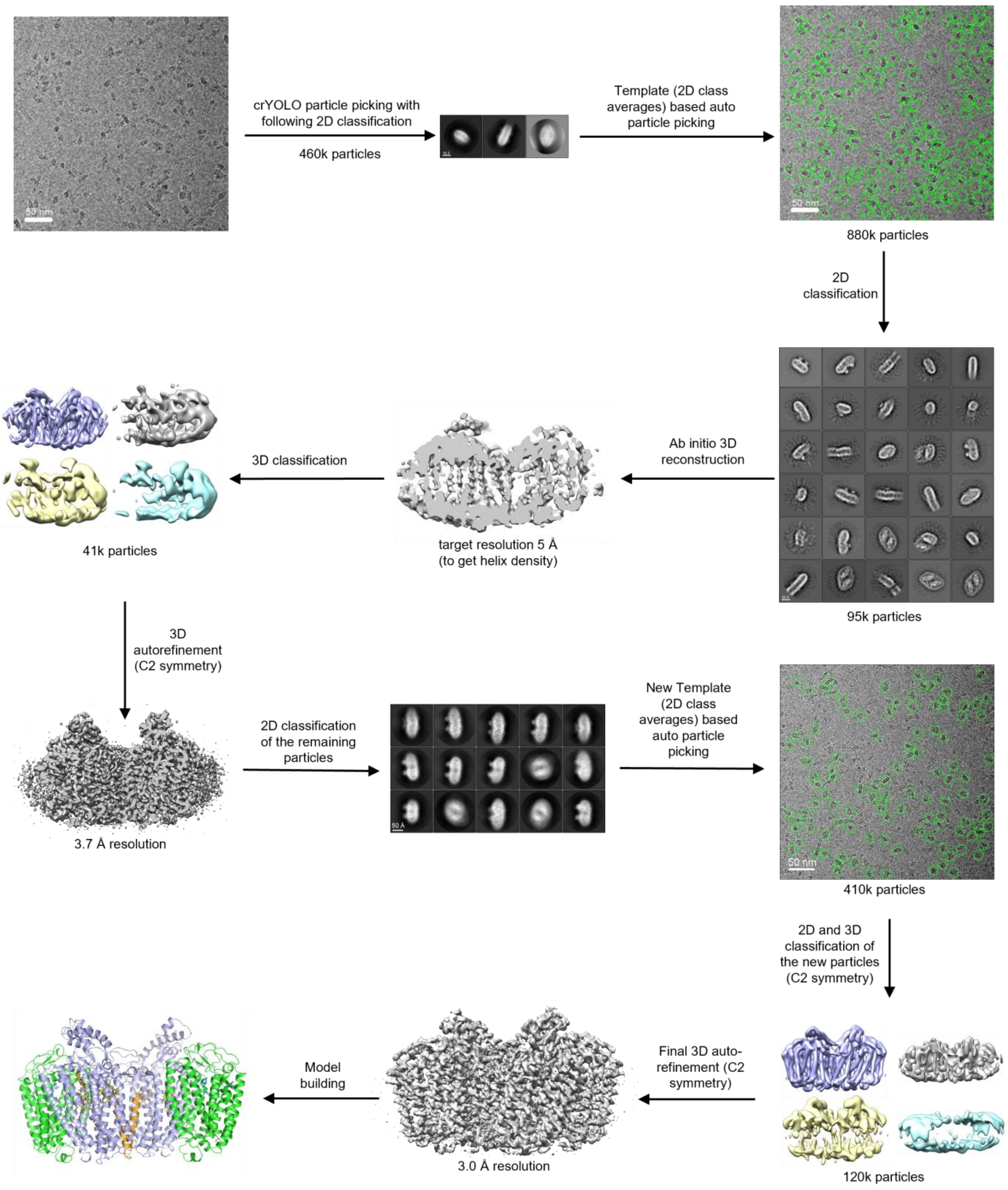

Supplementary Figure 4. Classification and refinement of the cryo-EM map for *E. coli bd*-II.

Convolutional neuronal network based particle picking of all micrographs was conducted with crYOLO[49] using the provided general network. The following workflow was conducted with Relion 3.1.0[50] to obtain the map of *bd*-II at 3.0 Å resolution. The micrograph is a representative of 35 micrographs.

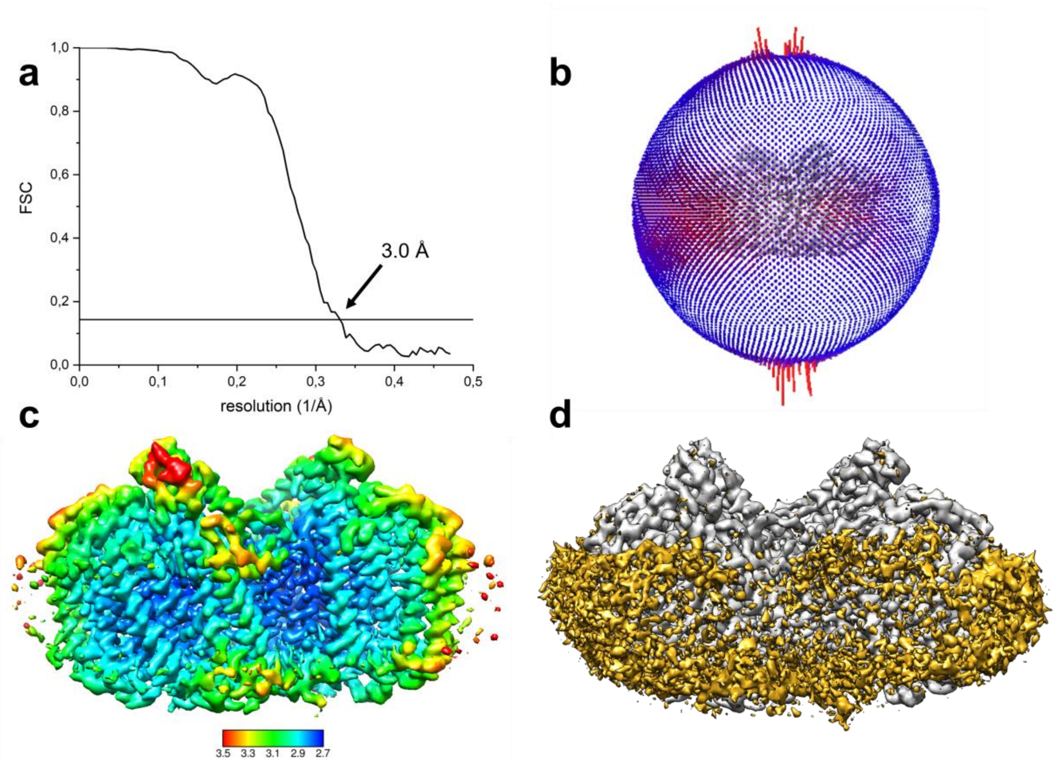

Supplementary Figure 5. Cryo-EM on *bd*-II.

a Fourier Shell Correlation plot with an overall resolution of 3.0 Å as determined according to the gold standard from independently processed half-sets as implemented in Relion 3.1.0[50]. **b** Angular distribution of particles contributing to the final reconstruction. **c** local resolution (Å) of the *bd*-II dimer map determined with the locres option in Relion postprocessing. **d** noise belt (yellow) around the protein map caused by the amphipol molecules that was not modeled.

a

Alignment of AppC/CydA/Gt CydA

```
Ec_bd_II      --MWDVIDLSRWQFALTALYHFLFVPLTLGLIFLLAIMETIYVVTGKTIYRDMTRFWGKL  58
Ec_bd_I       --MLDIVELSRLQFALTAMYHFLFVPLTLGMAFLLAIMETVYVLSGKQIYKDMTKFWGKL  58
Gt            MNGYDPVLLSRILTELTLTVHIIYATIGVGVPLMIAIAQWVGIRKNDMHYILLARRWTRG  60
                  * : ***     **    *:::. : :*: :::** : : : ...   *  ::: * :

Ec_bd_II      FGINFALGVATGLTMEFQFGTNWSFYSNYVGDIFGAPLAMEALMAFFLESTFVGLFFFGW  118
Ec_bd_I       FGINFALGVATGLTMEFQFGTNWSYYSHYVGDIFGAPLAIEGLMAFFLESTFVGLFFFGW  118
Gt            FVITVAVGVVTGTAIGLQLSLLWPNFMQLAGQVISLPLFMET-FAFFFEAIFLGIYLYTW  119
              * *..*:**.** :: :*:.   *   : : .*:::. ** :*   :***:*: *:*:::: *

Ec_bd_II      QRLNK-YQHLLVTWLVAFGSNLSALWILNANGWMQYPTGAHFDIDTLRMEMTSFSELVFN  177
Ec_bd_I       DRLGK-VQHMCVTWLVALGSNLSALWILVANGWMQNPIASDFNFETMRMEMVSFSELVLN  177
Gt            DRFENQKKHLLLLIPVAIGSSASAMFITMVNAFMNTPQGFELKNGE--LVNIDPIVAMFN  177
              :*: :   :*: :    **:**. **::*  .*.:*: *  . .:.      :    .    ::*

Ec_bd_II      PVSQVKFVHTVMAGYVTGAMFIMAISAWYLLRGRERNVALRSFAIGSVFGTLAIIGTLQL  237
Ec_bd_I       PVAQVKFVHTVASGYVTGAMFILGISAWYMLKGRDFAFAKRSFAIAASFGMAAVLSVIVL  237
Gt            PAMPTKVAHVLATSYMTSAFVLASIAAWHLWKGNRHIYHRKALHLTMKTAFIFSVASALV  237
              *.   .*..*.: :.*:*.*:.: .*:**:: :*.        ::: :      .     :.   :

Ec_bd_II      GDSSAYEVAQVQPVKLAAMEGEWQTEPAPAPFHVVAWPEQDQERNAFALKIPALLGILAT  297
Ec_bd_I       GDESGYEMGDVQKTKLAAIEAEWETQPAPAAFTLFGIPDQEEETNKFAIQIPYALGIIAT  297
Gt            GDLSGKFLAEYQPEKLAAAEWHFETSS-HAPLILFGTLEE-DNEVKYALEIPYALSILAH  295
              ** *.   :.: *   **** *  .::*.     *  : :..   :: ::    :*::**   *.*:*

Ec_bd_II      HSLDKPVPGLKNLMAETYPRLQRGRMAWLLMQEISQGNREPHVLQAFRGLEGDLGYGMLL  357
Ec_bd_I       RSVDTPVIGLKELMVQHEERIRNGMKAYSLLEQLRSGSTDQAVRDQFNSMKKDLGYGLLL  357
Gt            NHPAAVVTGLNDIPEDERPPL----------------------------------------  316
              .      * **:::   :     :

Ec_bd_II      SRYAPDMNHVTAAQYQAAMRGAIPQVAPVFWSFRIMVGCGSLLLLVMLIA-LVQTLRGKI  416
Ec_bd_I       KRYTPNVADATEAQIQQATKDSIPRVAPLYFAFRIMVACGFLLLAIIALS-FWSVIRNRI  416
Gt            ----------------------------YIHYLFDVMVTIGVFLMVVAAVYWLGSIFRWKW  349
                                          :.: * :**   * :*: :   :   : . :* :

Ec_bd_II      DQHRWVLKMALWSLPLPWIAIEAGWFMTEFGRQPWAIQDILPTYSAHSALTTGQLAFSLI  476
Ec_bd_I       GEKKWLLRAALYGIPLPWIAVEAGWFVAEYGRQPWAIGEVLPTAVANSSLTAGDLIFSMV  476
Gt            TAKNWFFGLLVAGGPLAMIAIAEAGWYLAEVGRQPWILRGYMKTAEGA--TTSAHVDTMLV  407
                :.*.:    : . **   **:****:::* ***** :    : *   .     *:..:   ::

Ec_bd_II      MIVGLYTLFLIAEVYLMQKYARLGPSAMQSEQPTQQQG-------- 514
Ec_bd_I       LICGLYTLFLVAELFLMFKFARLGPSSLKTGRYHFEQSSTTTQPAR 522
Gt            LFCLLYIVLVIASATVLIRMFRRNPVERELEERANRG---EVAP-- 448
              ::   ** ::::*.  :: :   * .*    :   .    .
```

Alignment of AppB/CydB/Gt CydA

```
Ec_bd_II      -MFDYETLRFIWWLLIGVILVVFMISDGFDMGIGCLLPLV---ARNDDERRIVINSVGAH  56
Ec_bd_I       -MIDYEVLRFIWWLLVGVLLIGFAVTDGFDMGVGMLTRFL---GRNDTERRIMINSIAPH  56
Gt            MTLEVIGISVLWLFL-----FGYIIVASIDFGAGFFSVYSHWANQQHILHRIIQRYLSPV  55
                ::    :  .:* :*      . : :  .:*:* * :         ::.   :**: . :.

Ec_bd_II      WEGNQVWLILAGGALFAAWPRVYAAAFSGFYVAMILVLCSLFF-----RPLAFDYRGKIA  111
Ec_bd_I       WDGNQVWLITAGGALFAAWPMVYAAAFSGFYVAMILVLASLFF-----RPVGFDYRSKIE  111
Gt            WEVTNVFLVFFFVGIVGFFPKT-----AYYYGSILLVPASIAIVLLAIRGSYYAFHTYGE  110
              *: .:*:*:     .:.. :* .      : :* :::** .*: :      *    : ::
```

```
Ec_bd_II      -DARWRKMWDAGLVIGSLVPPV---VFGIAFGNLLLGVPFAFTPQLRVEYLGSFWQLLTP   167
Ec_bd_I       -ETRWRNMWDWGIFIGSFVPPL---VIGVAFGNLLQGVPFNVDEYLRLYYTGNFFQLLNP   167
Gt            TERNWYL-LAYGL-TGLFIPASLSIVLTISEGGFVEESA----AGVALDYGKLF---ASP   161
               : .*      *:  * ::*     *: :: *.::          : : *   *    .*

Ec_bd_II      FPLLCGLLSLGMVILQGGVWLQLKTVGVIHLRSQLATKRAALLVMLCFLLAGYWLWVGID   227
Ec_bd_I       FGLLAGVVSVGMIITQGATYLQMRTVGELHLRTRATAQVAALVTLVCFALAGVWVMYGID   227
Gt            LSWSVVLLSVTSVLYISAVFLTYYADAAGD------EQARALLRRYA------LLWSGPT   209
              :     ::*:  ::  ...:*   : .  .       :  **:   .       :  *

Ec_bd_II      GFVLLAQ-DANGPSNPLMKLVAVLPGAWMNNFVESPVLWIFPLLGFFCPLLTVMAIYRGR   286
Ec_bd_I       GYVVKSTMDHYAASNPLNKEVVREAGAWLVNFNNTPILWAIPALGVVLPLLTILTARMDK   287
Gt            MLSALLIIYQLRYHNPEH-----YDNLW-------NVAWMLVISFLFFVITVWLLGRQRR   257
                            **         . *        : * :    ..  : . :     :

Ec_bd_II      PGWGFLMASLMQFGVIFTAGITLFPFVMPSSVSPISSLTLWDSTSSQLT-LSI-MLVIVL   344
Ec_bd_I       AAWAFVFSSLTLACIILTAGIAMFPFVMPSSTMMNASLTMWDATSSQLT-LNV-MTWVAV   345
Gt            FGWAFIALLFQYAFAFYAYGISHYPYLLYPYL------TIYDGFTNETMAMALIVAFIAG   311
               .*.*:   :     : : **: :*:::          *::*. :.:   : : :  :.

Ec_bd_II      --IFLPI-VLLYTLWSYYKMWGRMTTETLRRNENELY   378
Ec_bd_I       --VLVPI-ILLYTAWCYWKMFGRITKEDIERNTHSLY   379
Gt            LLLLIPSLYLLMRLFLFNKAYVKG---KWEGGKG---   342
                :::*   **   : : * : :      . .
```

Alignment of AppX/CydX/Gt CydS

```
Ec_bd_II      MWYLLWFVGILLMCSLSTLVLVWLDPRLKS-------   30
Ec_bd_I       MWYFAWILGTLLACSFGVITALALEHVESGKAGQEDI   37
Gt            MQTFLIMYAPMVVVALSVVAAFWVGLKDVHVNE----   33
              *  :  : . ::  ::..:. . :
```

b Alignment of AppC and AppB

```
APPC    MWDVIDLSRWQFALTALYHFLFVPLTLGLIFLLAIMETIYVVTGKTIYRDMTRFWGKLFG   60
APPB    MF--------------------------------------------DYETLRFIWWLLIG   16
         *:                                             *. :  :*  *:*
APPC    IN---------FALGVATGLTMEFQFGTNWSFYSNYVGDIFGAPLAMEALMAFFLESTFV   111
APPB    VILVVFMISDGFDMGIGCLLPLVARNDDERRIVINSVGAH--------------------   56
         :          *  :*:.  * :  : . :  :  *  **
APPC    GLFFFGWQRLNKYQHLLVTWLVAFGSNLSALWILNANGWMQYPTGAHFDIDTLRME-MTS   170
APPB    ------WEG--------NQVWLILAGGALFAAWPRVY-------AA-AF--SGFYVAMILV   93
               *:          .**:  *. * * *           :.  *  . : :  :
APPC    FSELVFNPVSQVKFVHTVMAGYVTGAMFIMAISAWYLLRGRERNVALR-SFAIGSVFGTL   229
APPB    LCSLFFRPLA-------------------------FDYRGKIADARWRKMWDAGLVIGSL   128
         :..*.*.*::                         :  **:  :.  *  :  * *:*:*
APPC    A-------IIGTLQLGDSSAYEVAQVQPVKLAAMEGEWQTEPAPAPFHVVAWPEQDQERN   282
APPB    VPPVVFGIAFGNLLLGVPFAFT----PQLRVEYLGSFWQLL-TP----------------   167
         .        :*.* **   *:       :::  : . **   :*
```

```
APPC      AFALKIPALLGILATHSLDKPVPGLKNLMAETYPRLQRGRMAWLLMQEISQGN-------   335
APPB      -----FPLLCGLL----------------SLGMVILQGG--VWLQLKTVGVIHLRSQLAT   204
               :* * *:*                :     ** *  .** :: :.   :
APPC      -REPH-V-----LQAFRGLEGDLGYGMLLSRYAPDMNHVTAAQYQAAMRGA---IPQVAP   385
APPB      KRAALLVMLCFLLAGYWLWVGIDGFVLLAQDANGPSNP--LMKLVAVLPGAWMNNFVESP   262
           *    *     * .:    *  *: :* .      *     :  *.: **       :*
APPC      VFWSFRIMVGCGSLLLLVMLIALVQTLRGKIDQHRWVLKMALWSLPLPWIAIEAGWFMTE   445
APPB      VLWIFPLLGFFCPLL------TVMAIYRGR---PGWGFLMASL-------MQFGVIFTAG   306
          *:* * ::     **      :::   **:     * : **            .  * :
APPC      FGRQPWAIQDILPTYSAHS--ALTTGQLAFSLIMIVGLYTLFLIAEVYLMQKY----ARL   499
APPB      ITLFPFVMPSSVSPISSLTLWDSTSSQLTLSIMLVIVLI-FLPIVLLYTLWSYYKMWGRM   365
          :   *:.: . :   *: :    *:.**::*::::: *   :: *. :* : .*    .*:
APPC      GPSAMQSEQPTQQQG   514
APPB      TTETLRRNENELY--   378
            .::: ::
```

c Conservation of amino acid residues in AppX and CydX

```
Appx      MWYLLWFVGILLMCSLSTLVLVWLDPRLKS
          *******:****************:****

CydX      MWYFAWILGTLLACSFGVITALALEHVESGKAGQEDI
          **************************** ********
```

Supplementary Figure 6. Sequence comparisons of *bd* oxidases.

a Alignment between the three homologous subunits of *E. coli* (Ec) *bd*-II, *bd*-I and *G. thermodenitrificans* (Gt) *bd* oxidase; *, identical, :, conserved substitutions, ., semi-conserved substitutions. The heme ligands (H19^{AppC}; H186^{AppC}, M393^{AppC} and E445^{AppC} and H99^{AppC} involved in substrate binding at heme *d*) are shown in red; residues of the Q-loop are shown in grey; L101^{AppC} inserted in the *E. coli* enzymes but not in *G. thermodenitrificans* and F104^{AppC} and I144^{AppC} providing a hydrophobic roof for oxygen binding are shown in green; W441^{AppC} needed for the formation of the F^{+*} reaction intermediate is shown in blue; V41^{AppC} and V42^{AppC} involved in the dimerization of *bd*-II are shown in dark blue; D239^{AppC} involved in Q and aurachin binding is shown in orange. Amino acid residues of AppB and CydB from *E. coli* and *G. thermodenitrificans* discussed to be involved in proton translocation[21,22,25] are shown in red. **b** Alignment between *E. coli* AppB and AppC. The sequence alignments were generated by ClustalW (www.genome.jp/tools-bin/clustalw) using the program default parameters. **c** Conservation of amino acid residues within the homologues of AppX and CydX. The conserved Leu residues are marked in red.

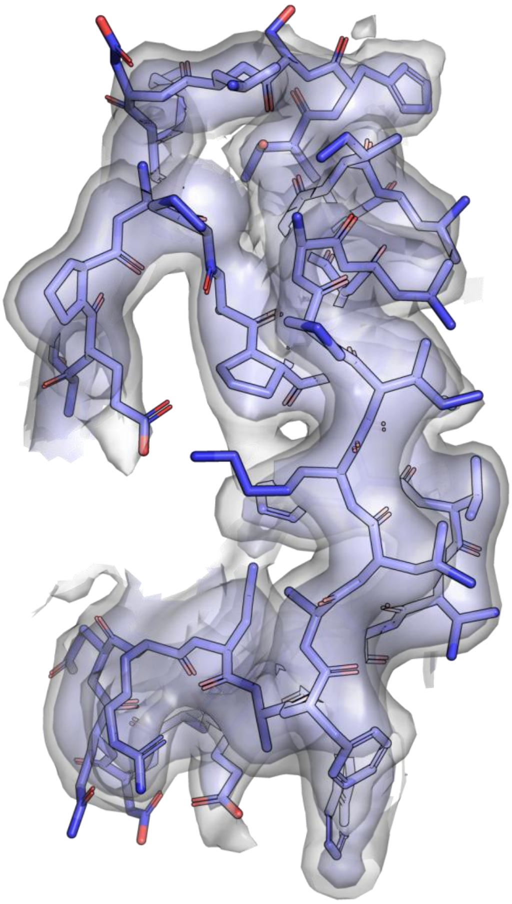

Supplementary Figure 7. Resolution of the Q-loop in the experimental Coulomb density map.

The map is shown as meshes, contoured at 1.5 σ (light blue) and 1.0 σ (light grey), both carved at 2.5 Å radius around model atom centres.

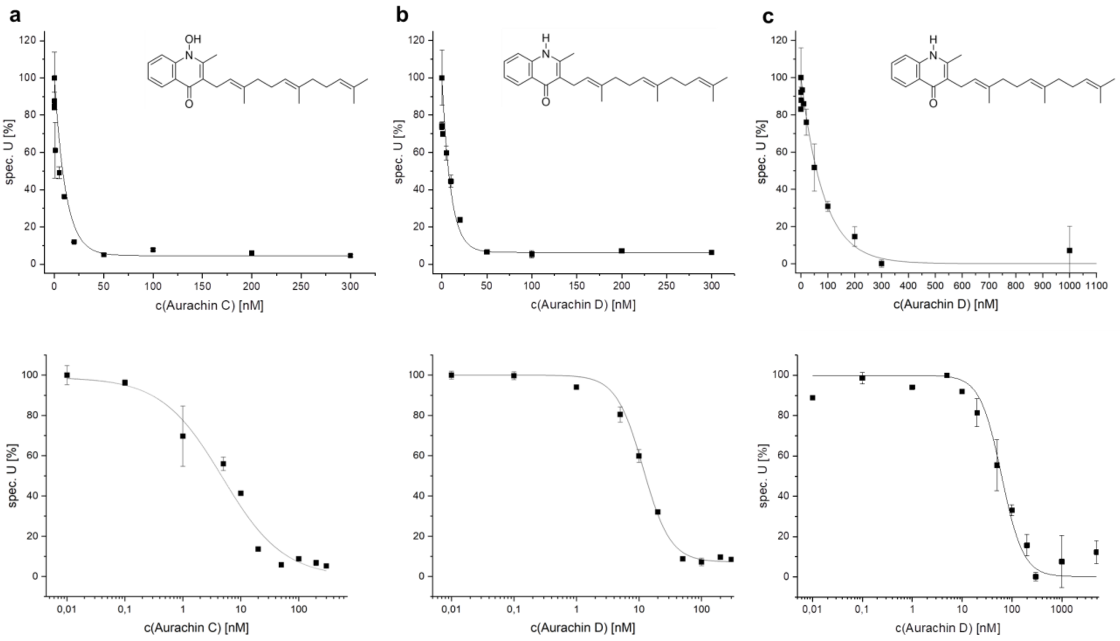

Supplementary Figure 8. Inhibition of *bd*-II by aurachin C (a) and aurachin D (b) and the D238N^{AppC} variant by aurachin D (c).

The upper curves show the direct inhibition plots and in the lower plots the inhibitor concentration is plotted on a log scale and the data points are fitted to a sigmoidal function to better visualize the inflection point. Inhibition of the duroquinol:dioxygen oxidoreductase activity of *bd*-II revealed IC_{50} values of 7.1 and 11.1 nM for aurachin C and aurachin D, respectively. The activity of the D239N^{AppC} variant was inhibited to 50% at 60.7 nM. Activities were determined from three biological replicates. Data are presented as mean values +/- standard deviations. The centres of the error bars indicate the unweighted mean values of the data points at a distinct aurachin concentration. Source data are provided as a Source Data file.

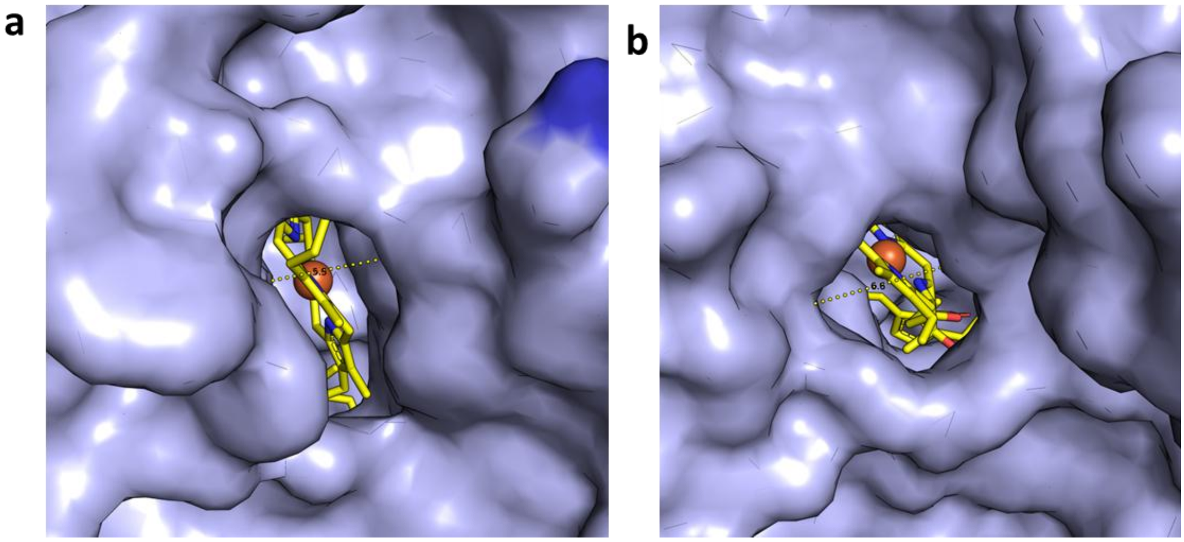

Supplementary Figure 9. Access to heme b_{595} in *E. coli bd*-II (**a**) and to heme *d* at the homologous position in *G. thermodenitrificans bd* oxidase (**b**, PDB: 5DOQ).
