# Supplementary material for: Structure of Escherichia coli cytochrome bd-II type oxidase with bound aurachin D
Source: Nat Commun. 2021 Nov 11;12:6498. doi: 10.1038/s41467-021-26835-2 (PMC8585947; doi:10.1038/s41467-021-26835-2)
Supplement: Supplementary file 3 — Reporting Summary [file 41467_2021_26835_MOESM3_ESM.pdf]

nature research

Corresponding author(s): Thorsten Friedrich

Last updated by author(s): Oct. 8, 2021

Reporting Summary

Nature Research wishes to improve the reproducibility of the work that we publish. This form provides structure for consistency and transparency in reporting. For further information on Nature Research policies, see Authors & Referees and the Editorial Policy Checklist .

Statistics

For all statistical analyses, confirm that the following items are present in the figure legend, table legend, main text, or Methods section.

n/a	Confirmed	
☐	☒	The exact sample size (*n*) for each experimental group/condition, given as a discrete number and unit of measurement
☐	☒	A statement on whether measurements were taken from distinct samples or whether the same sample was measured repeatedly
☒	☐	The statistical test(s) used AND whether they are one- or two-sided<br>*Only common tests should be described solely by name; describe more complex techniques in the Methods section.*
☒	☐	A description of all covariates tested
☒	☐	A description of any assumptions or corrections, such as tests of normality and adjustment for multiple comparisons
☐	☒	A full description of the statistical parameters including central tendency (e.g. means) or other basic estimates (e.g. regression coefficient) AND variation (e.g. standard deviation) or associated estimates of uncertainty (e.g. confidence intervals)
☒	☐	For null hypothesis testing, the test statistic (e.g. *F*, *t*, *r*) with confidence intervals, effect sizes, degrees of freedom and *P* value noted<br>*Give P values as exact values whenever suitable.*
☒	☐	For Bayesian analysis, information on the choice of priors and Markov chain Monte Carlo settings
☒	☐	For hierarchical and complex designs, identification of the appropriate level for tests and full reporting of outcomes
☒	☐	Estimates of effect sizes (e.g. Cohen's *d*, Pearson's *r*), indicating how they were calculated

Our web collection on statistics for biologists contains articles on many of the points above.

Software and code

Policy information about availability of computer code

Data collection	EPU v. 2.9 software on Titan Kryos G3 (300 kV)
Data analysis	EMAN, RELION v. 3.1.0, MotionCor 2 v. 1.0.5, crYOLO, CTFFIND v. 4.1, MolRep (from CCP4), Coot v. 0.8.9, MolProbity v. 4.5, Phenix v. 1.13, PyMol v. 2.4.1, Schrödinger software suite 2021-1 (PrepWizard, PROPKA v. 3.0), Desmond, Caver 3.0

For manuscripts utilizing custom algorithms or software that are central to the research but not yet described in published literature, software must be made available to editors/reviewers. We strongly encourage code deposition in a community repository (e.g. GitHub). See the Nature Research guidelines for submitting code & software for further information.

Data

Policy information about availability of data

All manuscripts must include a data availability statement. This statement should provide the following information, where applicable:

- Accession codes, unique identifiers, or web links for publicly available datasets
- A list of figures that have associated raw data
- A description of any restrictions on data availability

The cryo-EM maps of E. coli bd-II oxidase are deposited at the Electron Microscopy Data Bank (https://www.ebi.ac.uk/emdb) under accession number EMD-13048. The model of the bd-II oxidase structure was submitted to the worldwide Protein Data Base (https.//www.wwpdb.org/) with accession number 7OSE. The DALI database (http://ekhidna2.biocenter.helsinki.fi/dali/) was used to perform structural alignments with PDB entries with the accession numbers 5DOQ, 6RKO and 6RX4.

Field-specific reporting

Please select the one below that is the best fit for your research. If you are not sure, read the appropriate sections before making your selection.

☒ Life sciences ☐ Behavioural & social sciences ☐ Ecological, evolutionary & environmental sciences

For a reference copy of the document with all sections, see nature.com/documents/nr-reporting-summary-flat.pdf

Life sciences study design

All studies must disclose on these points even when the disclosure is negative.

Sample size	The number of cryo-EM particles in the dataset was the number of particles available.
Data exclusions	Particles that were not bd-II oxidase were excluded during analysis.
Replication	Single particle cryo-EM is based on averaging protein particles of more or less identical orientation in a vitreous thin ice layer. Accordingly, replication is not requiered to ensure statistical robustness of the data. In all structures obtained from the individual micrographs the general architecture of the E. coli bd-II oxidase remained identical. Thus, the data are considered as biological replicates of the presented structural data.
Randomization	No randomization is required for the experimental design of the study. Noteworthy, particles are randomized during data processing in Relion v. 3.1 (during 2D and 3D classification). Randomized half sets of particles are used in final reconstruction steps to determine gold-standard Fourier shell correlations based on the 0.143 level.
Blinding	The study was performed on a single enzyme sample. Therefore, the the experimental design did not require blinding approaches in order to ensure robustness of structural and biochemical data.

Reporting for specific materials, systems and methods

We require information from authors about some types of materials, experimental systems and methods used in many studies. Here, indicate whether each material, system or method listed is relevant to your study. If you are not sure if a list item applies to your research, read the appropriate section before selecting a response.

Materials & experimental systems

n/a	Involved in the study	
☒	☐	Antibodies
☒	☐	Eukaryotic cell lines
☒	☐	Palaeontology
☒	☐	Animals and other organisms
☒	☐	Human research participants
☒	☐	Clinical data

Methods

n/a	Involved in the study	
☒	☐	ChIP-seq
☒	☐	Flow cytometry
☒	☐	MRI-based neuroimaging
